# *The Sun Also Rises*: Tracing the evolution of humanistic values in anatomy pedagogy and research, including cadaveric acquisition practices

**DOI:** 10.1111/joa.13938

**Published:** 2023-07-31

**Authors:** Sourav Bhattacharjee, Sanjib Kumar Ghosh

**Affiliations:** ^1^ School of Veterinary Medicine University College Dublin Dublin 4 Ireland; ^2^ Department of Anatomy All India Institute of Medical Sciences Patna India

**Keywords:** Anatomy Act of 1832, body snatchers, Declaration of Helsinki, holocaust, humanism, informed consent, Nuremberg Medical Trial, Renaissance

## Abstract

Anatomy has always been at the intersection of the socio‐cultural and political landscape, where new ideas constantly replace older wisdom. From ancient Egyptians through the Greeks, and then the Romans, finally culminating into the European Renaissance—all the significant eras of human civilisation have left their insignia and distinct marks on the evolution of anatomical practices. Despite its utility as a tool for anatomy pedagogy and research that has proven its worth over millennia, cadaveric dissection has particularly been subject to political and social vicissitudes. A major debate about anatomical dissection lay with the ethical considerations, or its lack thereof, while acquiring corpses for demonstration in the dissection halls. From antiquity, anatomical dissection—often synonymous with medical studies—had typically been carried out on the dead bodies of executed criminals with certain laws, such as the Murder Act of 1752, facilitating such uses. Gradually, the uses of unclaimed bodies, resourced primarily from the impoverished sections of society, were also introduced. However, these body acquisition protocols often missed the crucial element of humanism and ethical considerations, while knowledge augmentation was taken as sufficient reasoning. Unfortunately, a gross disregard towards humanistic values promulgated heinous and illegal practices in acquiring corpses, including grave robbery and even murders like in the case of Burke and Hare murders of 1828. Follow‐up legislation, such as the Anatomy Act of 1832, and comparable laws in other European nations were passed to curb the vile. What distils from such a historical discourse on humane values in anatomy dissection, or medical science in general, is that the growth and integration of humanism in anatomy have never been linear, but there were intermittent and, yet, significant disruptions in its timeline. For example, there were serious human rights violations in anatomical practices during the Third Reich in Germany that perpetrated the holocaust. The medical community has kept evolving and introducing new moral values and principles while using such egregious events as lessons, ultimately resulting in the Declaration of Helsinki in 1964. This article revisits the heterogeneous journey of integrating humanistic values in anatomy practice. Such humanistic traits that, like medical science, have also developed over centuries through the inputs of physicians, researchers, and philosophers—from Greece to modernity with an important stopgap at the Renaissance—are a fascinating lore that deserves to be re‐envisioned through the lens of contemporary values and ethos. In parallel to human medicine, humanistic values continue to influence veterinary medicine, a welcome development, as our society condemns animal cruelty in any form. There are lessons to be learned from this historical journey of how humanism shaped many of the concepts that anatomists use now. Finally, and most importantly, it might prevent the medical community from repeating the same mistakes by cautioning against the traps that are there, and in a convoluted world where morality as such is eroding from our social fabric, will always be there. Such historical account acts as a righteous, ethical, and contextual compass to guide the existing and upcoming anatomists in discerning between light and dark, right and wrong, and roads—to be or not to be—taken.

## Graphical abstract

The Cave of the Hands (Cueva de las Manos) in Argentina, where the paintings of multiple human hands are visible. Considered an iconic symbol throughout the planet, it depicts a message from an unknown lost generation to us while symbolising the existence of humanity. This article traces the trajectory of humanism that has shaped anatomy teaching and learning over millennia, and the cave painting provides philosophical strength to the depth and scope of its discourse.

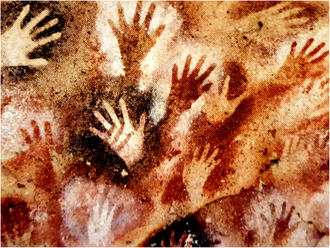

## 1 INTRODUCTION

The core principles of humanism existed in classical Greek philosophy, with the pre‐Socratic philosopher Protagoras (c. 490–420 BC) famously stating, “Man is the measure of all things” that was controversial for classical antiquity (Burrell, 1932) and was negated by many of the contemporary Greek philosophers, including Plato. A deeper preview of the Protagorian statement falls beyond the scope of this account, although it must be cautioned that such a thesis can sometimes be counterintuitive and vulnerable to individual interpretation.

It is undeniable that ancient Greek philosophy and society, in its evolution, started realising, expressing, and sporadically celebrating humane existence. The signature of embracing humans as the main pillar of being amidst the world's chaos riddled with wars and diseases symbolising death left abiding marks in Greek art and way of living. Despite raising quite a few eyebrows of the Puritans then (and possibly would have done the same even now), the human body became a form of exhibitionism with overt display in ancient Greece (Horton, 2015), particularly in art forms and athletics.

**FIGURE 1:**
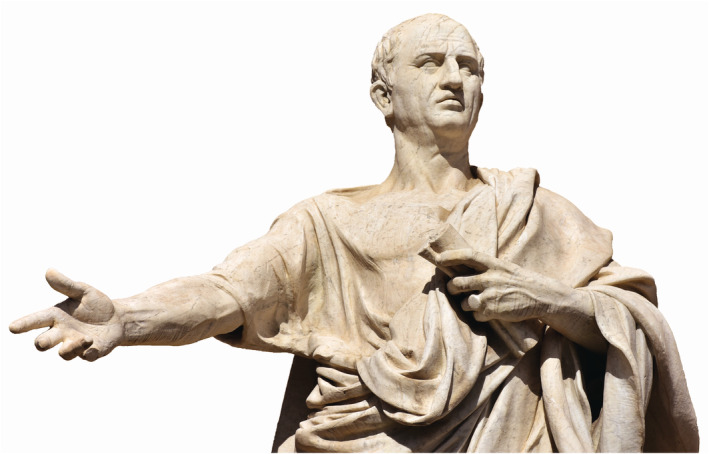
The bust of the Roman orator and philosopher Cicero (106–43 BCE), who is credited with having coined the term “humanitus”, which provided a conceptual framework for the amalgamation of humanistic values and ideas in the future.

With a gradual decline of the Hellenic empire and Rome set to dominate the power struggle in the occidental world, the societal context was conducive for then‐nascent humanistic concepts to crystallise and gain momentum. The Roman statesman, philosopher, and scholar Cicero (106–43 BCE), also known for influencing the burgeoning philosophy of the Renaissance and 18th‐century Enlightenment, is credited with having coined the Latin terminology “humanitus” (Figure 1). The scope of such humanism, or whatever primitive form of it existed in the Roman empire, was mostly confined within the premises of classical education. However, the hints of progressive liberalism in its primordial state started appearing with an advocacy of liberal education, open and didactic debates, and the right to quiz the *status quo*. The tenets of such humanistic views also identified a prerogative of individual equality and social rights that was subsequently followed by the concepts of democracy and secularism.

Humanism emerged as a rational philosophy informed by science, inspired by humanities, and motivated by compassion. It affirms the dignity of each human by promoting individual liberty and opportunity that resonates with the prevailing social structure and interpersonal relationships (Van Patten, 1975). Nonetheless, it is an integral component of modern education and aims to dissolve the artificial boundaries between stakeholders by fostering mutual trust and collaboration. To be precise, humanism seeks to balance cognitive and affective domains in learning (Stern et al., 2008).

Perhaps, understanding humanism in anatomy begins with the Renaissance (Summit, 2012). And it is not an overstatement. The European Renaissance that ushered into the Italian peninsula during the 14th century only to spread later through the continent provided an adequate template for what would come and shape human society in many ways. Some would argue that the Renaissance was fundamentally a cultural movement that once again brought Western society to a path of progress despite the fall of the Roman empire almost nine centuries back and sustaining multiple wounds of war, dissidence, and pestilence through the medieval dark ages. The Renaissance helped this cumulative social and cultural narrative evolve and, in some instances, mould itself into a rather unexplored landscape where humanism was poised to take centre stage (McManus, 2022).

With the appearance of polymaths and geniuses like Leonardo da Vinci (1452–1519) and Michelangelo Buonarroti (1475–1564), who embodied the archetypal “Renaissance Man”, the Renaissance helped increase the tolerance for humanism in society (Barnett, 2019). Despite a riveting tale, the causal factors of the Renaissance need separate space for discussion. Perhaps an escape during the Renaissance from the political upheavals and oppression by the papal hegemony in the middle ages delivered a fertile ground for revisionist ideologies to flourish and, most importantly, spread across Europe, including Germany, Belgium, England, and the Netherlands. A recovering economy with agrarian reforms and improvement in ploughing tools also brought prosperity to Europe.

The increased connectivity of Renaissance ideas, which started primarily as a Florentine spark with contributions from the Italian city‐states of Venice, Bologna, Genoa, Milan—and a shadow of its past but still functioning—Rome, encouraged young, bright minds to question the traditional power hierarchy and reject radicalism, including the tenets of Catholicism. An intense rivalry between these cities to outclass each other in creative excellence catapulted the quality of Renaissance art. The invention of printing press by Johannes Gutenberg in 1440 also accelerated the dispersion of knowledge and ideas among European philosophers (Dittmar, 2011). Furthermore, the fall of Constantinople in 1453 to the Ottoman Turks resulted in an exodus of scholars who took refuge in the Italian peninsula. The arrival of such a learned immigrant community of philosophers and researchers injected fresh blood into the European intelligentsia, already vibrating with the excitement of exploring new horizons.

**FIGURE 2:**
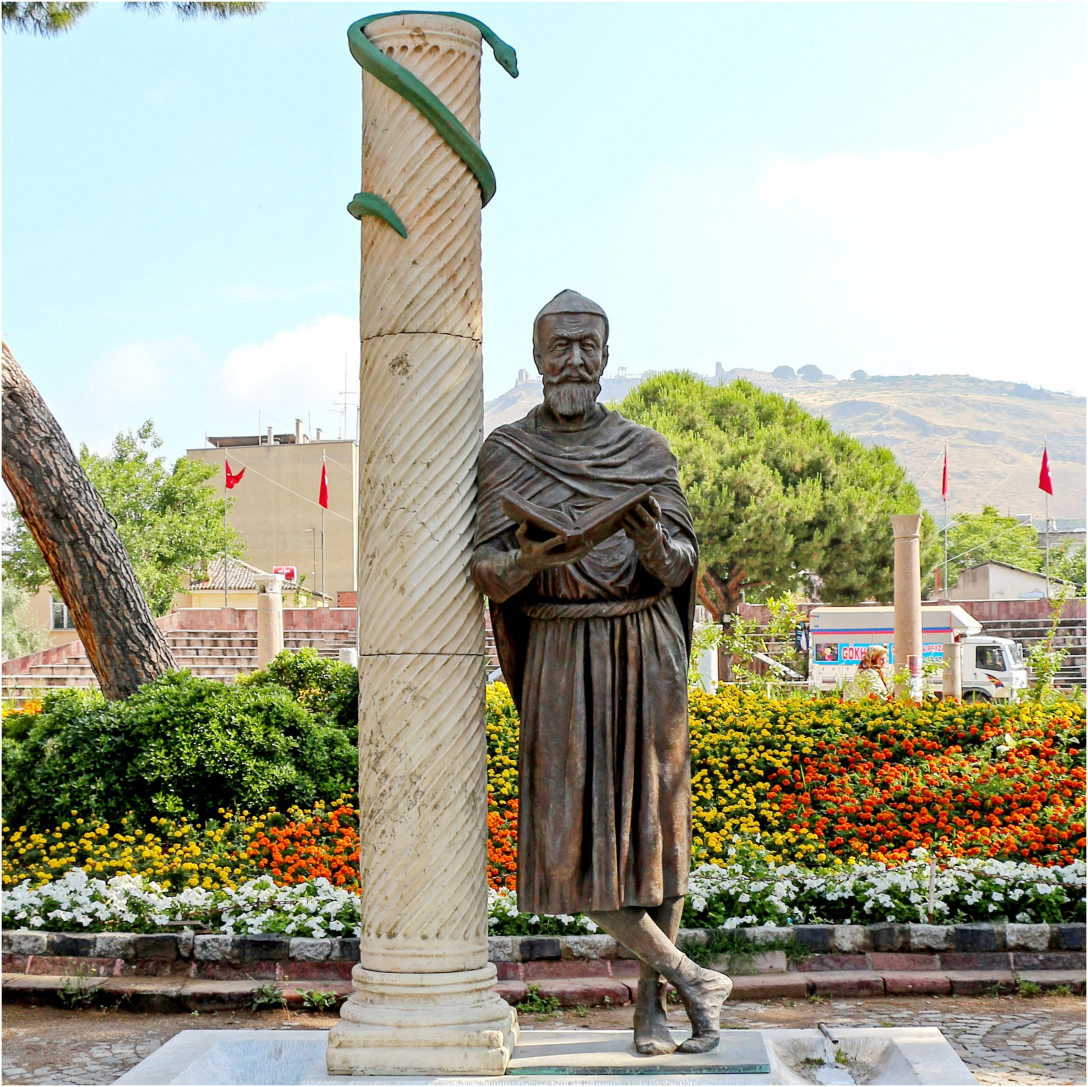
The statue of Galen in Bergama (Turkey). Figure reproduced from Wikimedia Commons.

An explosion of splendid artwork, literature, and music occurred during the Renaissance, while Renaissance creativity often depicted the human body with granular anatomical details. Naturally, knowledge of human anatomy emerged as a key requisite for expressing human body correctly and accurately in creative art forms, including sculptures and paintings. Thus, it is no wonder that almost all the Renaissance legends, including Leonardo da Vinci, Michelangelo, Donatello, Raphael, Botticelli, Titian, Tintoretto, and Caravaggio, took a keen interest in human anatomy (McMenamin, 2022). Perhaps rather than mere artists, it is time that we accept them as a golden generation of anatomists. Andreas Vesalius (1514–1564), often considered the founding father of modern anatomy, also made seminal contributions during the Renaissance (Xiang & Venkatesan, 2023) under his academic affiliations with various Italian universities, including Padova, Bologna, and Pisa. The work of Vesalius brought a seismic shift to the anatomic concepts laid by the Greek physician and surgeon Galen that used to dominate medical pedagogy until then (Figure 2).

**FIGURE 3:**
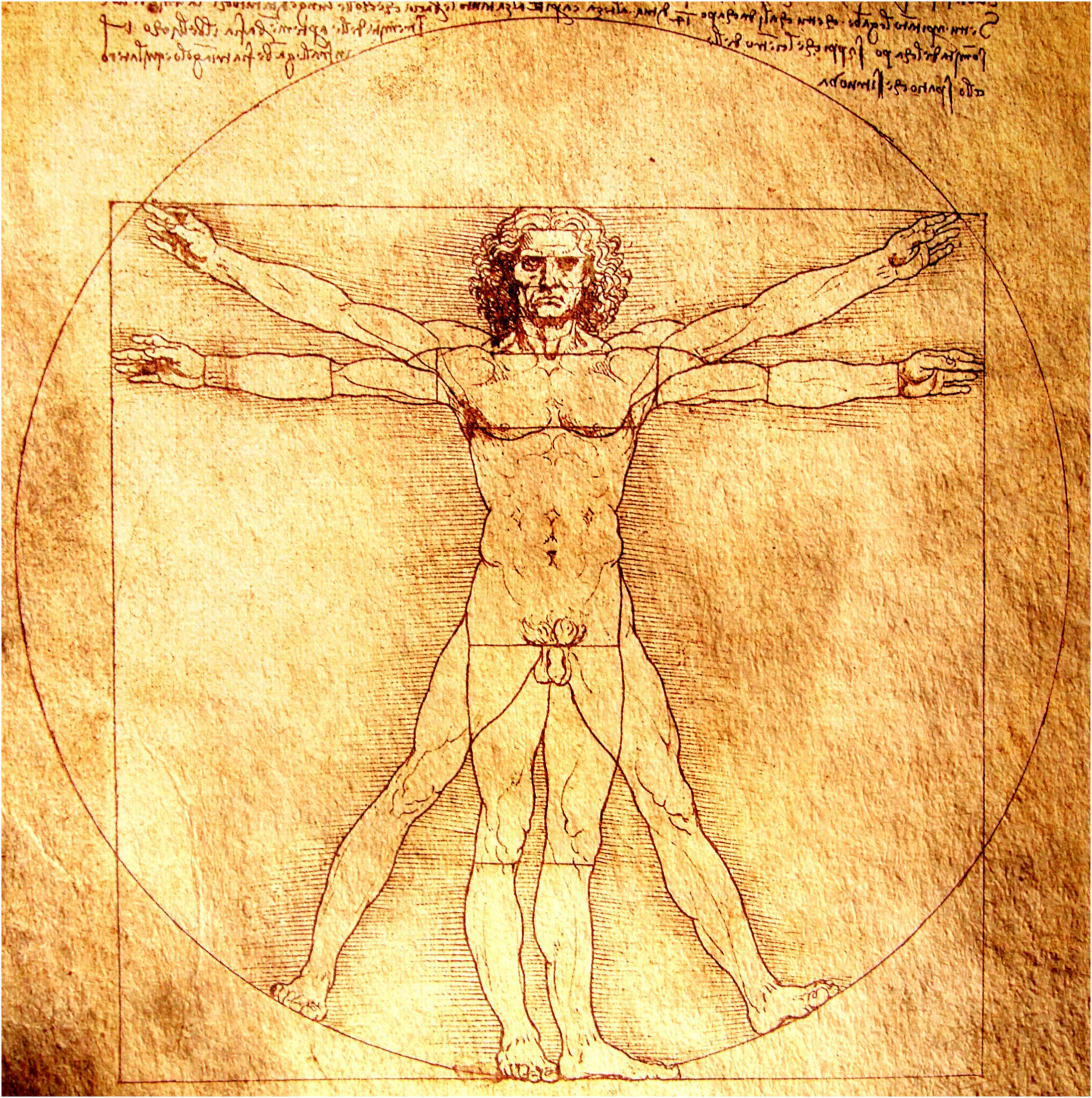
The Vitruvian man from the notebooks of Leonardo da Vinci.

Anatomy gave these talented people an intellectual haven to exercise their passion, express views, and collaborate. It is no wonder that some of the important artworks from the High Renaissance that have survived the test of time, such as the *Vitruvian Man* by Leonardo da Vinci (Figure 3), *David* by Michelangelo or Donatello, and *The Creation of Adam* fresco on the ceiling of Sistine Chapel in the Vatican City by Michelangelo, were rich in the exuberance of anatomical knowledge with perfection attained to a degree that seems unreal even today. In fact, Leonardo da Vinci was the first to draw a foetus *in utero* with meticulous detail of the umbilical cord and female external genitalia that could have easily put him in the league of blasphemous persons punishable by incarceration. He also theorised that foetal urine is excreted via the umbilical cord.

## 2 ANATOMY AT THE CULTURAL CROSSROADS

A rekindled interest in anatomy during the Renaissance (Andrioli & Trincia, 2004), from the vantage of scholarship, brought the discipline to the crossroads of intellectual and political debate, with the culturati now taking a keen interest in it. To an extent, anatomy has retained such a *suo generis* position even today, although the social context has indeed changed. Thus, anatomy as a discipline has undergone remarkable transformations throughout centuries that, more often than expected, were influenced by the principles of humanism (Dyer & Thorndike, 2000), which asked anatomists to ingrain a humanistic approach in their practices (Reidenberg & Laitman, 2002). It resulted in transitioning anatomy from a mere knowledge‐oriented discipline to a front‐runner in cultivating discipline‐independent skills guided by a set moral values of humanism (Štrkalj, 2014; Talarico, 2013). Inadvertently, incorporating the principles of humanism facilitated the process of reflection by considering the broader medical and socio‐cultural landscape (Bohl et al., 2011; Gregory et al., 2009).

A literature search relevant to the topic at hand from the following indexed databases, Medline and PubMed (United States National Library of Medicine); Scopus (Elsevier); Embase (Ovid Technologies, Inc.); CINAHL Plus (EBSCO Information Services); Web of Science (Clarivate Analytics); and Google Scholar (Google, Inc.), was conducted to gather a backdrop into the issue. Such browsing indicated that humanism in anatomy is intertwined with the history of anatomy as a discipline, particularly with the cascading events associated with sourcing human corpses and tissues. The principal factor that determined the fabric of humanism was the aspect of informed consent, or its lack thereof, of the donors whose mortal remains were used in enhancing anatomical knowledge. In this context, two pivotal events—the emergence of body donation programmes and the Declaration of Helsinki (1964) that helped formulate bioethics in medical sciences (Goodyear et al., 2007)—exerted a positive influence.

The landmarks in this road towards a well‐structured body donation programme as the principal source of human tissues were spread from the ancient period till the first half of the 20th century. Perhaps, such a humanistic approach got its springboard once the unfortunate events during the National Socialist Regime (Third Reich) in Germany ended. Despite a matter of the past now, the holocaust left a deep and indelible scar on humanism in medical science due to its gross disregard for individual consent while using humans for medical studies. It was also noted that in recent times, in addition to a strong emphasis on consent, a robust enhancement in academic activities in the relevant domain has led to the emergence of a multifaceted form of humanism in anatomy. Accordingly, the present account will focus on the chain of events resulting in the body donation programmes that eventually became the main, and often the sole source, of human tissues in anatomy pedagogy.

## 3 UNETHICAL USE OF HUMAN BODIES IN DISSECTION WITH A LACK OF HUMANISTIC VALUES

### 3.1 Corpses of executed criminals

Anatomy is one of the oldest elements of what the noblest profession embodies, while it is generally accepted that the practice of medicine during its onset in the ancient period was intricately associated, if not synonymised, with the pursuit of anatomical knowledge. Ancient scholars were well aware that to treat the human body as a physician, one must acquire a firm grip on its details (Loukas et al., 2016). Thus, began a long journey spread across millennia in search of anatomy knowledge (Ghosh, 2022). The primary concern at the very onset was access to the human body, which can be dissected to demonstrate its external and internal details, often to the students. The obvious choice was the mortal remains of a human being.

However, in ancient times, post‐mortem manipulation or dissection of a human body was considered sacrilege (Standring, 2016). Understandably, ancient scholars either performed unauthorised dissections incognito and away from the public eye due to fear of religious and social persecution or relied on royal patronage whereby the emperor would authorise the dissection of executed criminals, such as in Alexandria, Egypt (Brenna, 2022; Ghosh, 2015). Hence, in an unfortunate and paradoxical turn of events, the very onset of anatomical practice meant to enrich medical practice and better serve humanity deviated from humanistic traits by ignoring the individual consent of a person whose mortal remains were used for advancing knowledge. In a way, during the ancient period, anatomical studies were driven by the pursuit of knowledge with little or no focus on humanistic attributes.

In the middle ages, human dissection was forbidden by religious authorities in Europe to protect the priests and monks involved in the practice of medicine from coming in contact with human flesh and blood (Hernigou et al., 2021; Iorio, 2006). After a prolonged hiatus, human dissection resurfaced as the primary epistemological method to gain anatomical knowledge at the cusp of transitioning from the middle ages to the Renaissance (Ghosh, 2022). However, the same trend of dissecting bodies of executed criminals continued in Europe until the end of the 16th century (Hurren, 2013).

During the Renaissance, a quest for anatomical knowledge was no longer confined to physicians or medical students but also involved artists who tried to embody the details of human anatomy in their craft (McMenamin, 2022). Consequently, quite a few cases of human dissection conducted on bodies acquired through shady, illegal, and, at times, appalling means—with accusations framed against even the legends like Vesalius—were reported (Comer, 2022). In other words, the lack of humanistic approaches while resourcing human corpses or body parts to facilitate anatomy teaching and learning existed from ancient times to the Renaissance. As the scope of anatomical practice considerably increased during the Renaissance, the number of human dissections surged—and so did the malpractices—to stay relevant in this race (Ghosh, 2015). Hence, it is no wonder that the lack of humanistic traits in anatomical practice fulminated in this period.

### 3.2 The use of unclaimed bodies

From the onset of the 17th century, there was a sharp rise in the demand for human bodies across Europe, including Great Britain, due to the ever‐growing popularity of anatomy, with dissection emerging as the obvious, universal, and sole pedagogical tool (Brenna, 2022). This remarkable boost in scholastic interest in anatomy across Europe was due to the availability of popular anatomical texts imported from Italy, with a distribution made easy by the invention of the printing press (Ghosh & Kumar, 2019). The rising demand gradually snowballed into such uncontrolled momentum that by the mid‐18th century, laws were passed in most European countries to legalise dissection on the bodies of executed criminals (Hildebrandt, 2008). Notable among these legislations was the Murder Act of 1752 in England (Ghosh, 2015).

In subsequent years, encouraged by these legislations, the number of crimes punishable by hanging also increased. In fact, the legal system became so skewed in favour of human dissection that the discipline of anatomy became almost synonymous with capital punishment (Humphries, 2014). Despite these measures, the gap between the demand and supply of human corpses continued expanding. In response to the prevailing situation, many European nations, except England, legalised using unclaimed bodies of poor people, prison inmates, and patients in charitable hospitals or psychiatric asylums. Eventually, the use of unclaimed bodies outnumbered those of executed criminals (Magee, 2001).

**FIGURE 4:**
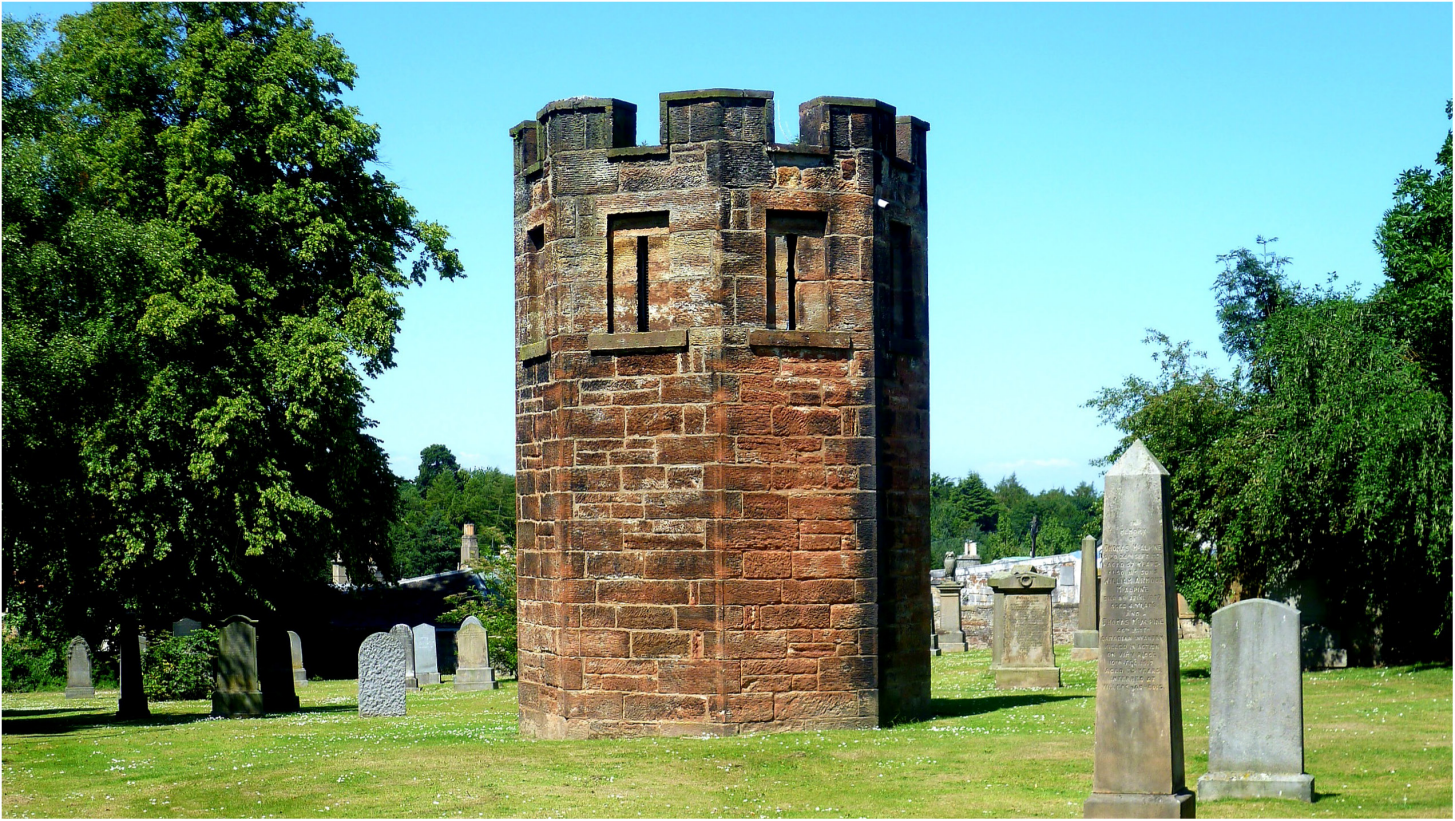
A watchtower of the Dalkeith Cemetery in Scotland, erected in 1827, to stop grave robbing.

In England, where the use of unclaimed bodies was illegal in the 18th century, the situation worsened rapidly by the early 19th century. Gruesome criminal acts, including grave robbing, body snatching, and even murders, were committed to supply bodies (Figure 4). Infamous among such dreadful incidents was the *Burke and Hare episode* (1828), which inspired the criminal gang of the *London Burkers*, who went out on a murder spree in 1831 (Mitchell et al., 2011). It would not be an overstatement that ignorance towards humanism in anatomical practice prevalent during the ancient period and Renaissance gradually transpired into abominable inhumanity by the early 19th century. During this time, a lack of humanism in anatomy and the seclusion of anatomists from larger society while dwelling in their ivory towers, with perhaps an element of arrogance, became obvious. Thus, rather than echoing their voice in line with the public disgust—anatomists, sometimes implicated in such crimes—referred to them as mere *resurrectionists* (Magee, 2001).

**FIGURE 5:**
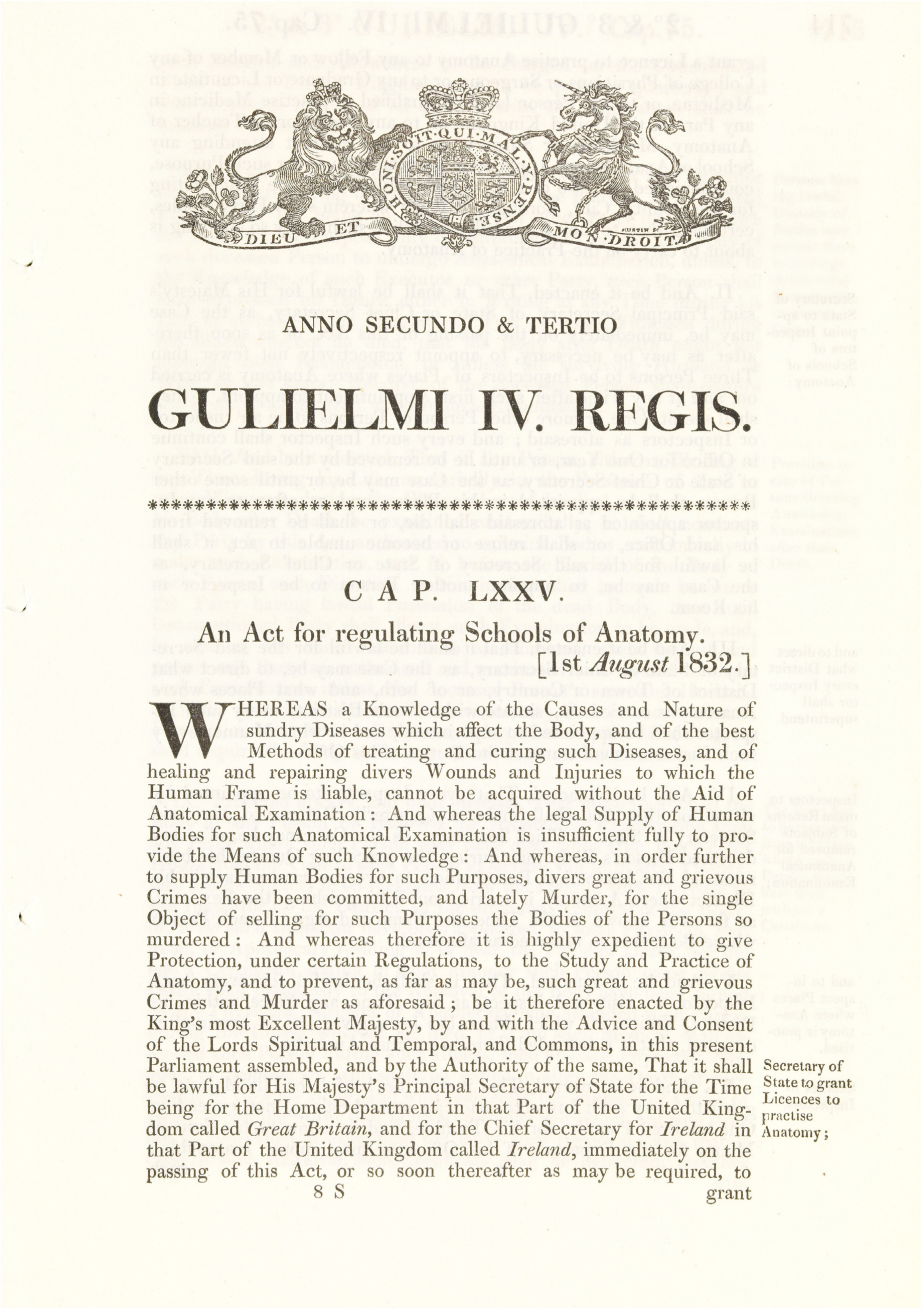
The cover page of the Anatomy Act of 1832.

Amidst growing public outrage and many deliberations, the British Govt. finally passed the Warburton Anatomy Act in 1832 (Figure 5, File S1). Like elsewhere in Europe, this act legalised the dissection of unclaimed human bodies, but unlike other European legislations, it prohibited the use of bodies of executed criminals (Harris, 1920; Kaufman, 2004). However, with a steady supply of unclaimed bodies now granted legal immunity, criminal means for acquiring human cadavers gradually got eliminated as it evolved into a business model that was neither sustainable nor profitable (Philp, 2022). Hence, the Anatomy Act remains an important landmark in the history of humanism in anatomy. It effectively curbed the century‐old inhuman tradition of dissecting bodies of executed criminals and, at least for the time being, gave breathing space to the administrative authorities dealing with a crisis.

In other words, the Anatomy Act (1832) was a glimmer of hope amidst the darkness that prevailed in anatomical practices during that period. Effective implementation of this act subsequently led to its adoption among the member states of the British Commonwealth (Ghosh, 2015). Despite its positive influence on anatomical practice, the Anatomy Act (1832) drew criticism for promoting the use of unclaimed cadavers and was perceived to be biased against poor people. Ironically, human dissection emerged from the shadows of capital punishment only to be used as a punishment for poverty, dubbed honestly, and in the absence of a better phrasing: *dissection for destitution* (Kaufman, 2005).

## 4 A CONTINUED LACK OF INFORMED CONSENT IN ANATOMICAL PRACTICE

Lawmakers of 19th century England, prior to the introduction of the Anatomy Act (1832), were concerned with mainly three aspects of anatomy dissection: (i) the fear in public regarding dissection with the very thought of their bodies going under the dissection knife instilling fear made worse with a slackness on the administrative front; (ii) the horrific crimes that were associated with the anatomical practice; and, (iii) a lack of a reliable source of human bodies that would also be acceptable to the society (Keith & Keys, 1957).

In a way, human dissection was frowned upon as a gruesome act and often interpreted as antisocial. The reported criminal acts further strengthened negative public opinion (Magee, 2001). Even the anatomists, at least a few, started voicing their concerns about the lack of humanity while resourcing human cadavers. Thus, the famous English anatomist William Harvey preferred to dissect his own family members rather than explore the mortal remains of a person arranged by individuals with dubious credentials (Philp, 2022). The seriousness of the prevailing situation in 19th century England can be gauged by the fact that John Abernethy, an eminent surgeon of the time, pleaded to the lawmakers to allow the use of unclaimed dead bodies for anatomical dissection. His justification was that this would establish a steady and legally sound supply chain of human bodies to accelerate scientific progress and serve humanity in the long run (Abernethy, 1819). Such views reflected the majority of the time, as it was perceived that the legalisation of using unclaimed bodies was the best solution. Most lawmakers also believed that using unclaimed bodies would liberate anatomical practice from inhuman activities and improve the public perception of cadaveric dissection (Kaufman, 2004).

## 5 PUBLIC PROTESTS AGAINST ANATOMICAL DISSECTION DRIVEN BY A LACK OF HUMANISM

By the mid‐19th century, the Anatomy Act and similar acts elsewhere in Europe, started taking effect in anatomical practice. From a broader perspective, implementing these legislations led to an apparent shift in the source of human bodies. Earlier, it was the prisons, which were now replaced by the hospitals where poor people were admitted for free medical care (Claes, 2018). However, these legislations largely failed to alter the public perception of human dissections, and regular protests became common.

These protests were primarily based on perceived injustice towards economically weaker sections of society, violation of local customs, and religious beliefs surrounding death. In some instances, they were also driven by political agendas (Moxham & Plaisant, 2014). The volume and intensity of such protests were so significant in England and the USA that they were referred to as the *Anatomy Riots* (Edwards, 1951; Magee, 2001). In sharp contrast, the situation was relatively peaceful in continental Europe due to supportive measures, such as an offer for a free burial of the dissected corpse in Germany, more societal acceptance for dissection of a deceased person availing state‐funded hospital care in France and Belgium, and a more tolerant sociopolitical climate in Austria (Claes, 2018).

## 6 THE EMERGENCE OF THE PRINCIPLE OF CONSENT IN MEDICAL SCIENCE

This prevailing sociopolitical narrative in Great Britain started evolving towards the late 19th century as the impact of the Anatomy Act (1832) and similar legislation started waning due to the shift in socio‐economic outlook of hospitals and medical care centres (Black, 2007; Zinner & Loughlin, 2009). Until then, services offered by these institutions were primarily availed by the poorer sections of society and served as a major source for the supply of corpses for dissection due to a relatively large number of unclaimed bodies. However, this impasse was broken during the latter part of the 19th century due to the introduction of health insurance, innovations in medical science, for example, the use of antimicrobials, and an apparent trend in hospitals tying up with universities in quest of transforming into teaching centres in parallel to caregiving, which led to a gradual softening of public opinion (Zinner & Loughlin, 2009).

Charitable hospitals, often transformed into prestigious medical centres by then, allowed the admission of paying patients. Consequently, the affluent section of society started receiving medical care in hospitals (Stolberg, 2016). Hence, the link between these hospitals and dissection rooms to supply bodies from an economically weaker section of society was now severed. During this period, the physician and the patients often belonged to the same or comparable socio‐economic stratum. Such a scenario led to the emergence of the principle of consent in the medical field, which focused on defining and, at times, regulating the doctor‐patient relationship (Bazzano et al., 2021).

In addition to the upgradation in the status of hospitals, legal references across late 19th century Europe also played a crucial role in ending the practice of dissection on unclaimed cadavers (Garment et al., 2007). The most notable was the *Pandectes belges*, where a parallel was drawn between the rights of the living and the dead. In a landmark judgement by the Belgian Supreme Court, it was ruled that an individual had the right to decide the fate of his/her post‐mortal remains (Claes, 2018). The judgement legally extended the rights of a living person even after death, whereby the post‐mortal body transformed from an object to an extension of the living person guided by the will of the deceased. In the purview of such legal referencing, the prevailing notion that society has the right to access the mortal remains of a poor person just because the subject availed social services in the form of free medical treatment during the lifetime lost traction (Tomasini, 2008).

Hence, medical institutions could no longer acquire unclaimed dead bodies of poor individuals for dissection. To be precise, the advent of consent due to an evolving profile of medical hospitals and existing legal provisions restoring the body's right exclusively to the concerned person rendered the use of unclaimed bodies untenable for medical utility. Consequently, and perhaps as an alternative sourcing strategy, medical schools started exploring the possibility of whole‐body donation with consent from potential donors and started focusing more on the doctor‐patient relationship (Mitchell et al., 2011).

A classic case may be mentioned here as testimony to the growing importance of consent in medicine during the early 20th century. The incidence was reported in 1923 from a hospital in Brussels, whereby the brain of Mme Lankester, a nurse in the hospital who died due to a neurological condition, was removed by neurologist Auguste Ley for research purposes (Boucher & Bouilliat, 1985; Claes, 2018). When Ley was charged with misconduct, he defended himself by stating that he had an *a priori* consent from the deceased. Ley also affirmed that as a treating physician, he considered it appropriate to give due importance to the patient's wishes above existing regulations (Claes, 2018). His arguments reflected the cultural importance of an individual's consent in medical science during that period.

As the support for individual consent to determine the fate of their bodies increased steadily, the 19th‐century notion of providing medical care in exchange for human bodies ended (Stolberg, 2016). Thus, anatomists increasingly had to comply with the principle of consent. It is also apparent that the principle of consent prepared the ground for formal willed body donation programmes, which in due course emerged as the main source of cadavers for dissection in European and American medical schools from the 20th century (Ciliberti et al., 2020; Garment et al., 2007).

## 7 THE SOCIAL REFORMS PROMOTED BY CONSENSUAL BODY DONATION

In alliance with religious authorities, the evolution of social attitude towards death from the second half of the 19th century also contributed to the eventual introduction of body donation campaigns (Saunders, 2009). In a nutshell, the orthodox outlook towards death and a dead body, which existed since the ancient ages, gradually shifted towards a more progressive mindset. This was evident as even while combating the morbidity surrounding the death bed of an individual, rational Christians, Deists, sceptics, and atheists alike sought to demystify death while promoting a frank outlook regarding the fate of the physical remains of a deceased individual (Porter, 2000).

Several luminaries from different sections of society, including philosophers, medical practitioners, social workers, politicians, and religious functionaries, significantly contributed towards a modified outlook on death and promoting body bequeathal (Ghosh & Walocha, 2022). The contribution of English philosopher and social reformer Jeremy Bentham (1748–1832) may be mentioned in particular. He was the proponent of *utilitarianism*, which conceptualised that an individual's greatest happiness is linked to a large number of people (Martin et al., 2021). In other words, his theory reflected the essence of altruism, a selfless concern for the well‐being of others, which constituted (and still does) the prime motivation behind body donation.

In those days, religious customs created a potential inhibition towards body donation. Traditional methods for a funeral were accepted norms, and society at large was apprehensive at the thought of mortal remains going under dissection (Garment et al., 2007). Bentham's theory rejected such religious taboos involved with the disposal of mortal remains of a person. Instead, it highlighted the benefit to society from such acts. In a way, his theory gave more weightage to the service of humanity than individual rights (Spector, 1963) and pioneered the core principles of body donation. After he died in 1832, as per his wish, the body was donated for anatomical dissection at University College London. His mortal remains were preserved as an “auto‐icon” that acted as a memorial and continues to be displayed at the entrance of the student centre (Marmoy, 1958; Philp, 2022).

Bentham's theory of utility and his altruistic act of body donation inspired many to imitate the noble and selfless act for the progress of science (Richardson & Hurwitz, 1987). By the turn of the 20th century, public opinion about anatomical dissection was shaped by stories of doctors and public figures who donated their bodies (Hutchinson et al., 2020). The role of scientific and learned societies is also worth mentioning. The French *Societe d'autopsie mutuelle* promoted body donation by emphasising eternal existence after death in scientific findings allied to societal progress (Claes, 2018).

The works of Susan Lederer, an expert in medical history, highlighted the importance of anatomists in the USA donating their bodies more frequently during the early 20th century in transforming the mindset and attitude of society towards body bequeathal programmes (Lederer, 2019, 2020). Such selfless acts and visionary efforts from custodians of medical science, often physicians, were pivotal in the evolution of the social impact of anatomy, including its shift from involuntarily acquired corpses to voluntarily donated human bodies for dissection.

From the early 20th century, body donation emerged as a prominent resource for anatomy dissection. Initially, awareness building was achieved at the level of a doctor‐patient interface, and the significance of a human body was confined within a clinical setup (Garment et al., 2007). Despite several positive developments, the body donation programmes had a lacklustre start due to a dearth of concrete legislation, which made body donation legally complicated (Ghosh, 2015). In particular, getting the mortal remains after death became increasingly difficult because the deceased's relatives/next of kin often refused to hand them over despite a prior pledge in place. As a remedial measure, legislation such as the Uniform Anatomical Gift Act was introduced in the USA in 1968 and was amended in subsequent years (Dalley et al., 1993).

Similarly, the British government passed the Anatomy Act (1984) and the Human Tissue Act (2004) to streamline the process of body donation (Greene, 2003; Taylor & Wilson, 2007). The European Federation of Experimental Morphology recommended similar measures in 2005, which have since been incorporated within legal provisions and implemented by European medical schools (McHanwell et al., 2008). The gradual flow of events from the late 19th century eventually led to the establishment of body donation as a prominent and often exclusive source of human cadavers. This sequence of events leading to body donation in anatomical practice reinstated the concept of humanity in the discipline.

## 8 THE GROSS DISREGARD OF INFORMED CONSENT DURING THE THIRD REICH: AN EVIL IN ACTION

The old world is dying, and the new world struggles to be born: now is the time of monsters.

—Antonio Gramsci (1891–1937)

**FIGURE 6:**
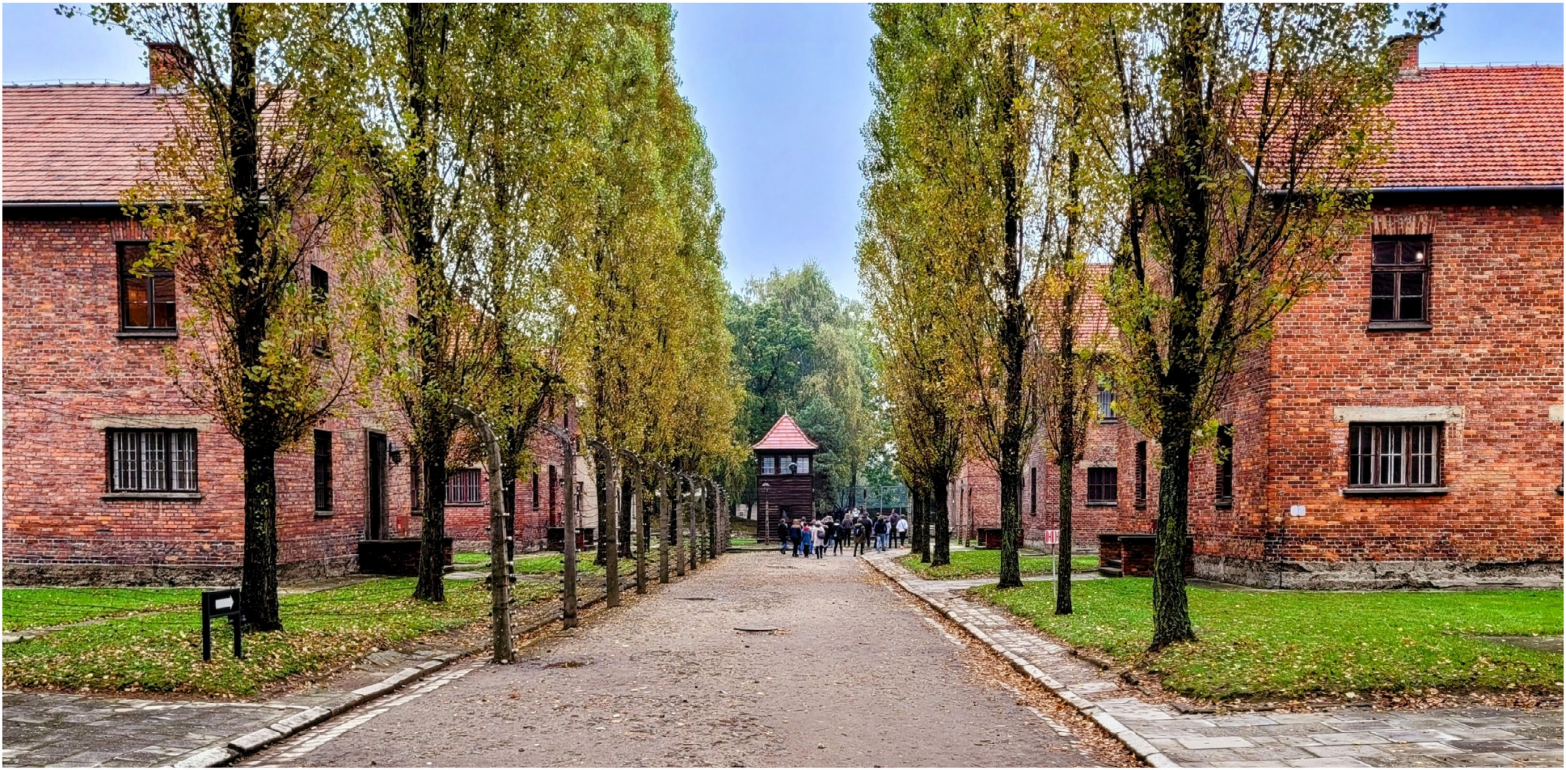
The barracks of the Auschwitz Concentration Camp in Poland where an unknown number, probably in many thousands, of mostly Jewish prisoners, were exterminated by the Nazis guided by their “final solution”. Figure from the personal collection of Sourav Bhattacharjee.

Atrocities committed on human lives during the National Socialist Regime/Third Reich in Germany (Figure 6) constituted a dark chapter in human history (Czech, Druml, et al., 2021; Czech, Weindling, & Druml, 2021). The practice of medicine and related research activities during this period, often unceremoniously referred to as “Nazi medicine”, involved malicious experiments with a blatant disregard for human rights (Hofer et al., 2020). Accounts recorded by Holocaust survivors and proceedings from the Nuremberg Medical Trial chronicled repulsive details of extreme cruelty inflicted upon human lives (Bruns & Chelouche, 2017).

During this period, the four major areas that experienced a violation of ethical principles in medical research were as follows: (i) gross neglect regarding the aspect of informed consent as political and war prisoners were coerced to participate in medical experiments; (ii) total disregard for the suffering of individuals; (iii) urge to pursue for and establish new scientific knowledge led to ignorance of any respect for individuals; (iv) taking full advantage of establishments that evaded public scrutiny, such as concentration camps, mental asylums, and state‐run hospitals, for unethical experiments that were given legal immunity (Roelcke, 2004; Weindling et al., 2016). The unethical and inhumane treatment of human lives flourished with support from a totalitarian regime and an ignorant scientific community turning a blind eye to heinous events (Eckert et al., 2018). The most unfortunate aspect of this saga was that there were attempts to justify these actions during the Nuremberg Trial in the name of scientific knowledge advancement and societal well‐being (Roelcke, 2004).

The revelations and observations about the Third Reich were a reality check and eye‐opener for the scientific community regarding the possible threats to human lives in the name of unregulated state‐sponsored medical experiments (Nunes et al., 2019). The Nuremberg Medical Trial strongly suggested setting clearly defined boundaries for research involving humans (Marrus, 1999). A considerable amount of introspection and deliberation went on in the scientific community, and an outline of ethical principles governing human experiments emerged: (i) seeking explicit informed consent from any potential participant should be mandatory; (ii) a participant should be informed about the possible outcome of the study; (iii) a participant should be regarded as a partner in the study rather than a subject; (iv) during the study, participant interests should be given full respect under best available medical knowledge; (v) impetus for advancement in existing knowledge should not override a participant's free will and well‐being; (vi) violation of research regulations should be punishable by forceful sanctions, and where applicable, prosecution (Roelcke, 2004; Weindling et al., 2016).

Like any other discipline in medical science, anatomy was also adversely affected by ethical malpractices during the Third Reich (Aumüller & Grundmann, 2002). Human tissues obtained from executed political and war prisoners from the Nazi period were used in anatomical studies by institutes in Germany and Austria (Freilinger et al., 2022; Oehler‐Klein et al., 2012). Revelations were made in 1989 that academic Institutions in West Germany, particularly the Institute of Anatomy of the University of Tübingen and the Max Planck Society Institute of Brain Research in Frankfurt, possessed specimens harvested from holocaust victims. In Tübingen, the Institute of Anatomy held specimens prepared from the mortal remains of hundreds of political prisoners, mostly Jews, executed in Stuttgart. The Max Planck Society Institute for Brain Research specimens included cerebral tissues obtained from the victims of infamous Nazi “euthanasia” killings. These neurological specimens included those of children killed in special institutions and adult patients subjected to euthanasia in psychiatric hospitals.

The preserved specimens were identified and buried as part of a planned programme in Tübingen and Munich—the administrative centre of the Max Planck Society—in 1990 (Seidelman, 2012). The burial programmes were followed by memorial ceremonies, which allowed the scientific community to reflect on the cruelty inflicted on human lives during the Third Reich and the deep scars such atrocities left (Weindling, 2012). Similar disclosures were made regarding the Institute of Anatomy at the University of Vienna (Czech & Brenner, 2019). A suspicion was raised in 1994 about the presence of Nazi insignia in the signatures of the illustrators Erich Lepier and Karl Endtresser in the renowned anatomical work “Pernkopf Atlas of Anatomy” (Czech, Druml, et al., 2021; Czech, Weindling, & Druml, 2021). The atlas emanated from the institute, and a substantial part of the work was carried out during the Third Reich. Unsurprisingly, the presence of Nazi insignia, and other related pieces of evidence that surfaced in due course, led to the realisation that dissected human bodies of executed political or war prisoners were included in the atlas (Hildebrandt, 2006). It was also divulged that hundreds of decapitated cadavers and severed heads, possibly also resourced during the Third Reich, were retained by the institute.

Two other infamous collections were unearthed at the Vienna Museum of Natural History and the Vienna Psychiatric Hospital. The Vienna Museum had anthropological collections, including plaster‐of‐Paris life masks prepared from Jewish victims and skeletal specimens prepared from mortal remains of Polish prisoners of war (Seidelman, 2012). The Vienna psychiatric hospital collected brain specimens of child victims killed during the Nazi euthanasia programme (Neugebauer & Stacher, 1999). Following the publication of an investigation report in 1998, which documented the presence of human tissues of Holocaust victims in these institutes, all such specimens were identified, removed, and buried respectfully. The entire process of isolating these specimens and their burial was completed by March 2002 (Czech, Druml, et al., 2021; Czech, Weindling, & Druml, 2021; Seidelman, 2012).

The events that transpired during the Third Reich regarding macabre atrocities inflicted on human lives in the name of medical experiments and scientific studies are a lesson for any discipline (Pringle, 2010). Pursuing scientific knowledge and related experiments will be considered useful only if it follows moral principles and the outcome benefits humankind (Hildebrandt, 2022). The subsequent developments and deliberations after the fall of the Nazi regime emphasised the importance of defining limits in any scientific practice and respecting ethical boundaries. The inhumanity perpetrated by the Third Reich constituted a dark but watershed event in the history of medical science, including anatomy, as the preventive measures that came into effect after the regime's fall were critical in humanising medical research. Imbibing the attributes of respect and dignity towards human lives should be the core element of any scientific advancement. Cultivating humanistic traits among custodians of the discipline is critical for instilling moral principles among the present generation so that good examples are left behind for them to emulate and, perhaps, serve as a source of pride and inspiration.

## 9 THE DECLARATION OF HELSINKI (1964): EMERGENCE OF BIOETHICS

The outline of ethical principles in research involving human individuals that emerged from deliberations within the scientific community, catalysed by the proceedings of the Nuremberg Medical Trial, was considered during the drafting of the Nuremberg Code (Barondess, 2000). The code comprised a set of principles for human experimentation that was initially created as a memorandum on the 9th of August 1947. The points in the memorandum were reiterated by the judges in the verdict passed against Rudolf Brandt and others (Nuremberg Medical Trial), convicted of Nazi atrocities (Katz, 1996). The verdict passed on the 20th of August 1947 approved the original six points in the memorandum, and additional points were added as per the medical experts consulted as an advisory panel. A ten‐point document finally emerged, which came to be known as the Nuremberg Code (Weisleder, 2022).

The code included principles such as informed consent, absence of force or coercion, beneficence towards participants, and rationally designed scientific experiments (Merz, 2018). It was a strong reprimand for any disrespectful and unethical treatment of humans in the name of scientific experiments and is widely regarded as a significant document in the history of clinical research ethics (Ghooi, 2011; Markman & Markman, 2007). Soon after the Nuremberg Code was introduced, the World Medical Association was founded on the 18th of September, 1947.

Following the principles and points included in the Nuremberg Code, a draft proposal was submitted to the Medical Ethics Committee of the World Medical Association in 1953. The draft was published a year later as the “Resolution on Human Experimentation” (Rice, 2008). One notable deviation in the World Medical Association resolution from Nuremberg Code was the scope of informed consent. The resolution proposed that in case of participants incapable of giving informed consent, their legally authorised representatives may do so on their behalf. Thus, informed consent was rationally modified to give a little leeway to researchers (Diekema, 2006). This initial draft of 1954 was presented as a first draft declaration by the Medical Ethics Committee in 1961 (Ballantyne & Eriksson, 2019). Finally, it was adopted unanimously at the 18th general assembly of the World Medical Association in Helsinki (June 1964). The document, titled “Recommendations guiding doctors in clinical research”, came to be known as the “Declaration of Helsinki” (File S2). It was the first internationally recognised set of ethical principles for research involving humans (Rohrich, 2007).

In the years to follow, the guidelines in the original Declaration of Helsinki (1964) and its subsequent amendments emerged as the most influential charter on ethical practices in medical research and continue to be so even today (Shrestha & Dunn, 2020). It was a major boost towards a re‐emergence of humanism in anatomical practice after it got decimated during the Third Reich. The declaration emphasised the significance of informed consent, which gained momentum with the body donation campaigns of the first half of the 20th century, but was defenestrated during the Third Reich. Upon adopting the Declaration of Helsinki (1964), the strong focus on informed consent prepared a stable ground for humanism in medicine to flourish.

## 10 HUMANISM AS PART OF MODERN ADVANCEMENTS IN ANATOMICAL EDUCATION

### 10.1 Recognising the contribution of body donors

Human tissues are the backbone of anatomical practice, and donated cadavers constitute an essential anatomy teaching and learning component. The selfless act of body donation is critical for the progress of medical science and is a priceless service to humanity (Ghosh, 2017a; Orsini et al., 2021). It is a matter of regret that the valuable contribution of body donors is seldom recognised in the scientific literature, which remains ironic (Bolt et al., 2010). A group of Editors‐in‐Chief from seventeen anatomical journals published a set of recommendations in a recent article to address this area of concern. The principal recommendation constituted a uniform code of conduct whereby the author(s) should recognise the contribution of body donors and express their gratitude. The statement explicitly mentioned that anatomical research could be undertaken because of the kind act of body donation (Iwanaga et al., 2021). These recommendations are a welcome measure in anatomical sciences and respect body donation (Riederer, 2016). Embracing the published recommendations will constitute good practice in anatomical research and set a good example for future researchers.

### 10.2 Increasing awareness about equality, diversity, and inclusivity in anatomy

Equality, diversity, and inclusivity (EDI) is a term that refers to the representation and participation of different groups of individuals, including, but not limited to, people of different ages, races, gender orientations, and ethnicities (Gill et al., 2018). It is fast gaining popularity among academicians, and anatomy as a discipline is no exception. The concepts apply to all entities where the eventual outcome depends on the cumulative contribution of the relevant stakeholders (Harrison‐Bernard et al., 2020). Therefore, embracing the virtues of EDI is critical for the functioning of institutions, societies, associations, periodicals, and other functional academic cohorts.

In other words, EDI is a key component for all the pillars of an academic discipline (Tzovara et al., 2021). The American Association for Anatomy (AAA) Board of Directors in 2021 constituted a task force to study the effects of structural racism on its membership. Accordingly, the task force's initial report was published in 2022, which observed that African Americans were underrepresented in the AAA compared to their US population. The recommendations of the task force were bi‐pronged: (i) to acknowledge the finding and issue a statement accepting responsibility; (ii) to support positive measures which are already underway to improve the existing scenario (Sumner et al., 2022).

Another article focussed on the need for enhanced communication regarding EDI within the learning environment. The article highlighted a training series implemented in the College of Graduate Studies and Arizona College of Osteopathic Medicine, Arizona, USA. The series involved a communication‐based exercise between the faculty members and first‐year medical students through a discussion on issues relevant to EDI. The essence of this programme is appropriately reflected in the title of the course (“Humanity in Medicine”), which it is a part of (Muldoon, 2022).

Such communication centred around the concept of EDI contributes to building the professional identity of healthcare personnel, enabling them to connect with society better. Considering the trend of increasing global diversity in the educational sector, promoting values coherent with the virtues of EDI has become an essential norm (Rosenkranz et al., 2021). Imbibing such values would enable individuals to develop an inclusive view of society. It would also assist them in achieving professional development with advancement of the academic environment (Bennett, 2021). The current emphasis on EDI issues within the anatomical sciences is critical for the humanistic outlook of a discipline that primarily deals with human tissues.

### 10.3 Encouraging the use of gender‐neutral language

Historically, the language used in anatomical practice carried gender bias (Draper, 2021). Initially, the interpretation of human anatomy was based on a unisex model focusing more on the male anatomical details. With a gradual change in perceptions and the emergence of a broader societal outlook, a binary model was adopted with comparable emphasis on both the male and female anatomical details (Evans, 2013; Kachlik, 2021). However, a considerable gap still exists towards achieving gender neutrality regarding the language used in anatomy. To be more precise, transgender and non‐binary gender identities are often ignored (Ahmad et al., 2019), which goes against the values of EDI.

Moreover, empirical research has linked this aspect with inadequate and unrealistic patient information, leading to a negative attitude and adverse behaviour among healthcare providers (Goldhammer et al., 2018). Hence, the issue of gender bias in the language of a discipline has a significant bearing on the professional outlook of future clinicians. In this context, an article recently published by the AAA argued in favour of incorporating inclusive language in anatomy education. It is believed that using gender‐neutral language within the curriculum would contribute towards establishing transgender and non‐binary identities before future health professionals, fostering improved professionalism.

The article provided useful recommendations for implementing desirable modifications in existing anatomical language. The authors also highlighted the impact of a language inclusive of gender‐neutral pronouns in anatomical education (Easterling & Byram, 2022). The transition proposed by the authors in the mentioned article is essential for an overall enhancement of anatomy education (Štrkalj & Pather, 2021; Taylor et al., 2018), which would be useful for medical students to assimilate knowledge unblemished of gender bias. Recognising the transgender and non‐binary identities in society through the lens of anatomy delivered with respectful and inclusive language would be useful for patient care.

### 10.4 Emphasis on the ethical use of human tissues

Conducting scientific research within ethical norms is now considered a good practice for any discipline, including anatomy (Ghosh, 2020). Watchdogs are active now at every level of scientific activity (e.g., institutional boards, scientific societies or associations, journals, and conference secretariats), and they have been successful in keeping cases of ethical misadventures on the lower side (Comer, 2022; Cornwall & Hildebrandt, 2019). However, there is no better custodian of ethics in research than the researchers themselves. In other words, for a coherent adoption of ethical standards across the discipline, anatomists themselves have to adopt a proactive role in maintaining the highest ethical standards (Jones, 1998). Such recommendations for the ethical use of human tissues for anatomy research were proposed by twenty‐two Editors‐in‐Chief of anatomy journals from seventeen countries.

It was a much‐needed initiative in this context, and the efforts of such a diverse group of eminent researchers uniting under one umbrella are commendable. The documentation of the recommendations in an established journal provided an impetus for the cause and added to the impact of the message. In the recommendations proposed for anatomical research involving human tissues, the author(s) should include a statement while undertaking research activity affirming that all ethical guidelines and legal aspects were followed (Iwanaga et al., 2022). The success of this novel initiative depends on anatomists embracing the recommendations.

Adherence to ethical standards is an integral parameter now to assure quality research. Moreover, as secondary data in the form of systematic reviews and meta‐analyses are on the rise, a breach of ethical norms will have long‐term implications (Sataloff et al., 2021). Ethics in anatomy is an essential dimension and contributes to the outlook and public perception. Thus, any research output today is viewed through the prism of ethical principles, and the prospects of any scientific discipline will inevitably be determined by the ethical standards followed during its execution.

### 10.5 Supporting students psychologically to reduce mental distress during dissection

The issue of students experiencing mental distress during human dissection has been an active area of research. Medical students are often exposed to human remains for the first time in the dissection hall, which has been acknowledged to be a significant life event for them, leading to an emotionally stressful situation (Böckers et al., 2012; Boeckers et al., 2010). It has been reported that coping mechanisms come into effect during this time, enabling most students to adapt positively (Anyanwu, 2015). However, around 4%–6% of students experience adverse effects, such as nightmares, insomnia, and learning difficulties (Boeckers et al., 2010).

Such psychological issues associated with dissection may resemble post‐traumatic stress disorder and even impact their future professional lives (Chang et al., 2018). The initial response to this issue was to reduce dissection hours or conduct dissection with other modes of teaching and learning. However, such a response resulted in compromised anatomical knowledge and adversely affected the quality of clinical training (Boeckers et al., 2010). With human dissection recognised as a valuable educational tool, measures to curtail contact hours were also dismissed (Ghosh, 2017b). Hence, anatomy educators mostly focussed on dealing with the issue retrospectively based on student experiences (Cheng et al., 2013).

A recent publication tried to address the issue prospectively and reported a positive outcome. This study hypothesised that dissection‐associated mental distress is an “anticipatory phobic reaction” before initial contact with the human remains. A lack of adequate and accurate information regarding human dissection might precipitate it. Therefore, a psychological training module was designed and applied to the first‐year student cohort, with the outcome analysed prospectively. It was found that students exposed to the pre‐dissection psychological training module handled dissection‐associated mental distress better, engaged more in the learning process, participated in dissection, and performed better academically than the control group (Chaudhuri, 2022). The model described in the study may be worth emulating as mental distress associated with human dissection is a valid concern also from an academic vantage (Ford et al., 2018). It may inflict long‐term mental scars on students and stunt their professional growth. Simultaneously, it has a bearing on learning outcomes as affected students may exhibit diminished motivation towards dissection; thus undermining the impact of a valuable learning tool (Daya & Hearn, 2018).

## 11 HUMANISM AS PART OF MODERN ADVANCEMENTS IN VETERINARY EDUCATION

With an increasing emphasis on the One Health preview of diseases (Bhattacharjee et al., 2022), veterinary anatomy education and research have started integrating humanistic values (Desmond, 2022). Maybe it is fair to state that with time, society has started realising the value of life, in all its speciation, which ultimately leads to building a robust ecosystem where humanity thrives. Humankind needs other life forms to ensure its existence, and the veracity of such an argument is imprinted in history when humanity succumbed to many waves of pandemics caused by zoonotic diseases, including the latest SARS‐CoV‐2 pandemic. Unfortunately, extreme animal rights violations—including culling, medical experimentation, indiscriminate use for entertainment purposes, and food sourcing to feed an already over‐populated world—to name a few, have continued for centuries. The veterinary medical schools of the Western nations have made great strides, especially in the last few decades, to incorporate ethical practice into their curricula, where budding veterinarians are made increasingly aware of their responsibilities in handling animal patients with care and compassion.

Scientific research has slowly but steadily accepted that the intrinsic value of animal life is no less than that of humans. In other words, animal sentience should not be used to justify cruelty against them. Thus, rather than treating animals with ulterior motives for financial gain, they should be respected as stakeholders in our social well‐being. The regulatory authorities and institutions providing veterinary education, such as the American Veterinary Medical Association, Federation of Veterinarians of Europe, European Association of Establishments for Veterinary Education, and Royal College of Veterinary Surgeons, now strongly recommend the integration of ethical practices, guided by humanistic values, as one of the desired *Day One Competencies* in veterinary graduates. Student knowledge and competencies in ethical practices are checked in licensing examinations, such as the North American Veterinary Licensing Examination (Hernandez et al., 2018).

Project proposals involving animal experimentation are now evaluated through the lens of 3Rs, viz., Replacement, Reduction, and Refinement, before being approved (Hubrecht & Carter, 2019). Institutions of higher research, including universities, especially in the occidental world, have often installed their own research ethics review committees (Pietrzykowski, 2021), and the reviewing is quite strict. The researchers proposing animal use need to convince the ethics committee that they have taken all possible alternatives to animal research into account while the benefits from the project outweigh the animal discomfort and associated loss of lives.

Operative procedures on animals, such as vivisection (Festing & Wilkinson, 2007) and neutering (Downes et al., 2015), are also on the ethical radar, and such surgical procedures undergo stringent monitoring. Similar ethical approvals are now required for resourcing animal cadavers for dissection purposes. Like humans, informed consent on behalf of pet owners is now essential for body acquisition. Euthanistic practices, however, continue to be controversial (Knesl et al., 2017; Persson et al., 2020), and while the debate on whether they should be banned altogether or like humans should only be conducted under extenuating circumstances continues, as of now, they need ethical assessment.

## 12 FUTURE PERSPECTIVES

A good rule of thumb is ‘Biology enables, culture forbids’. Biology is willing to tolerate a very wide spectrum of possibilities. It's culture that obliges people to realise some possibilities while forbidding others.

—Yuval Noah Harari, Sapiens: A Brief History of Humankind

If there is one thing that is clear from the history of humanism in anatomy education and research, that is interference from the existing sociopolitical system, while many a time, the decision‐making provision was confiscated from the anatomists. Sadly, as a community, anatomists also allowed it to happen for sometimes trivial gains or maybe vested interests. Thus, the Third Reich could continue their malevolent acts with vocal support or silent inaction—that could be construed as support—from the medical community.

There were indeed doctors who tried to save human lives, such as Gisella Perl (1907–1988) or Eugene Łazowski (1913–2006), with whatever resource they possessed. Three Italian doctors, Vittorio Sacerdoti, Giovani Borromeo, and Adriano Ossicini, even invented a spurious disease called “Syndrome K" to safeguard Jewish people from the Nazis. However, we miss evidence that physicians, including anatomists, as a community, stood against such a brutal regime and protested in one voice. Unfortunately, the reality was the opposite: Josef Mengele and his band of thugs, regrettably also often doctors, literally enjoyed a free ride over the dumps of corpses.

**FIGURE 7:**
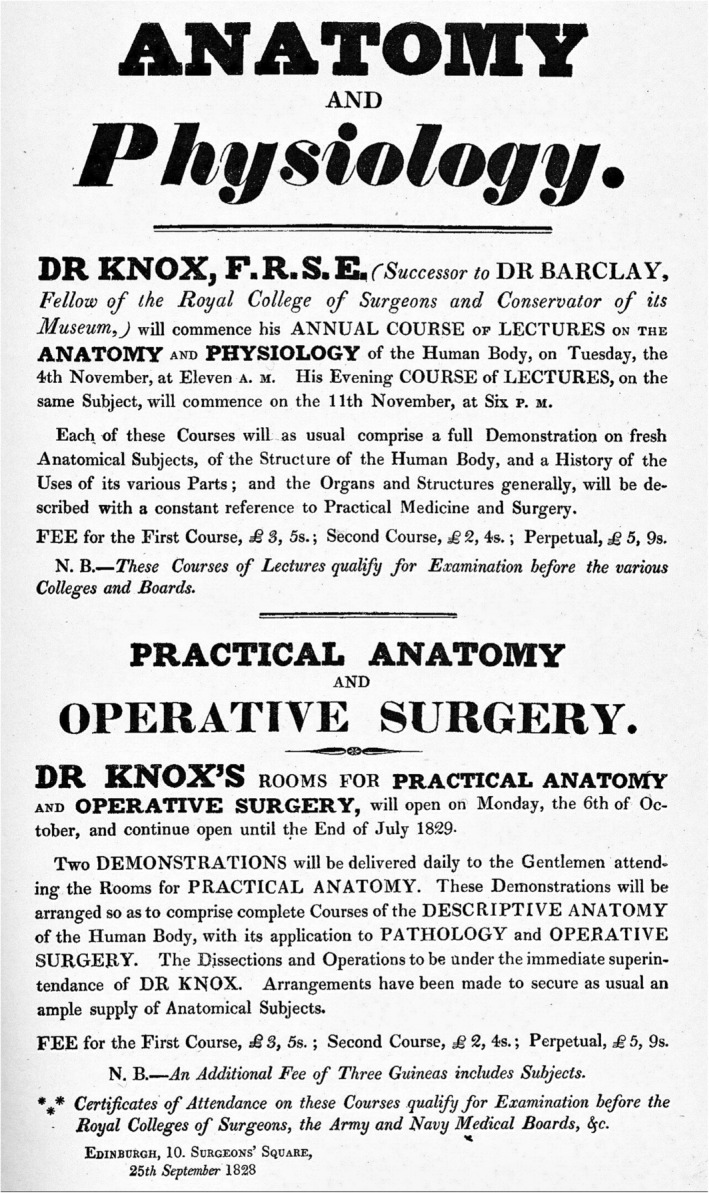
The handbills distributed by the surgeon Robert Knox involved in the Burke and Hare murders.

Similarly, in the days of the Burke and Hare murders (1828), one must not forget the surgeon Robert Knox who was an accomplice to the grotesque crimes. In his advertising handbills for upcoming dissection sessions (Figure 7), there used to be a clear mention, and we are quoting verbatim:

Each of these courses will as usual comprise a full Demonstration on fresh Anatomical Subjects, of the structure of the Human Body, and a History of the Uses of its various Parts; and the Organs and Structures generally, will be described with a constant reference to Practical Medicine and Surgery.

The handbills even quoted prices for attending these lecture courses: £3.5 s for the first one, £2.4s for the second, and £5.9s for perpetual attendance. Finally, the pamphlet, of course, did not forget to allure the students by conveying an eternal assurance that is difficult for them to ignore, “*These Courses of Lectures qualify for Examination before the Colleges and Boards*.” What seems extraordinary is that despite the authorities, including the Royal College, fully aware of the ongoing crisis of resourcing human cadavers, did not find it strange, notice any red flags, or even feel any need to inquire about such “incredible” confidence in Robert Knox to announce finding, “*fresh Anatomical Subjects*” in each course. Nonetheless, the crime continued in open daylight and at the behest of medical institutions.

Perhaps, it is difficult to protest against the “system”, especially when it stands unapologetic and brazen with a loaded rifle. However, the medical community cannot desert the larger society where hardworking people pay taxes and fund our medical education with the hope that the students will emerge not only as good physicians but also form enlightened minds who will feel their agony and share their frustration. Society expects the anatomists to stand against despots or power‐hungry cabals for whom human lives mean little. The public respects anatomists, physicians, or surgeons because they believe in their intellect and look up to them as torch‐bearers of a just society that will lead to a better tomorrow and make this world a better place.

In a way, the anatomists, as part of the larger medical fraternity, are expected to demonstrate the highest humane standards and set examples for others to follow. With this belief, a person enters a body donation programme trusting the anatomists will use the post‐mortal remains to serve humanity. The conviction to help others even after death gives a purpose to a living donor. Such noble gestures must be respected. In understanding and taking ownership of their responsibility as anatomists, the future of humanism in anatomical practice will thrive. Anatomy, thus, is not only a subject to master to become a physician but often societal and, at times, philosophical rearing of an individual.

It remains debatable if the power hierarchy of occidental nations has changed much from what it used to be in the middle ages or if it has become more Machiavellian with better skills in disguise. Today, war, racial violence, genocides, civil unrest, suicides, mental diseases, drug addiction, child abuse, pandemic, climate change, and many more maladies, including brazen hypocrisy, continue to widen the existing fissures in our society. As a species, humans are not more united now but rather increasingly fragmented with intolerance towards others along the lines of gender, race, opinion, belief, and many other issues. Unfortunately, the media narrative often adds fuel to the fire rather than salvaging these societal fault lines. An uncontrolled, unregulated, and unmonitored explosion of social media spreading all sorts of lies, trolls, and disinformation amplifies the miseries. The ludicrous thing in this insane mud‐pelting on social media, with its undeniable nuisance value that keeps a large audience entertained and hitched to it, is that each warring faction claims the other one to be radical, sometimes as radical as the Nazis, while portraying themselves as genuine truth seekers.

A functioning dissection hall is not immune to such battlegrounds in a social crucible. It is time again that anatomists unite and state unequivocally that they are not a party to this evil, nor do they buy any of the justifications used to create this environment of mutual distrust and fear. Rather than slicing society and instigating peril, anatomy teaches how similar humans are—in blood, flesh, nerves, bones, and other fillers of existential template—that humanity badly needs. If the anatomists and the larger medical fraternity remain silent today, not only will they lose the entrustment of the people, but also society will perhaps never forgive them. We are now at a humanistic crossroad where society and anatomists need each other, and anatomists should grab this opportunity. Let *Carpe Diem* be our maxim.

Throughout the history of humanity, including the two world wars fought in the last century, the medical fraternity and anatomists have sacrificed incredibly to serve people, which cannot be denied. Despite growing challenges, including inflating class sizes and shrinking resources, anatomists have prepared doctors who have treated ailing patients, and they (anatomists) deserve respect for their service. The reality, unfortunately, is often the reverse, where anatomists now need to take lectures from those who are sometimes oblivious to the dissection hall and have never been involved with medical studies in any capacity. Today's anatomists are passed sermons packaged in abstract buzzwords nicely scribbled in various policy documents.

Amidst this uproar, the actual anatomy education gets diluted, while the essence of knowing the human body as a foundation stone of medical education remains neglected. The university campus seems different where like everything, the dissection hall is also noted as a “facility”, and students evaluate a degree based on whether it is “worth the money”. Despite being registered charities, many universities pay obscenely high salaries to the top brass in management circles, whereas teachers—who prepare professionals for society—are on the streets demanding a basic wage. Like charity begins at home, humanism begins with treating people who serve society with respect. Writing policy documents might be the first step and necessary, but what matters is to abide by those policies, which, unfortunately, is often missing. Similarly, expecting humanism in the dissection hall while the anatomists are treated disrespectfully and unfairly will never work.

As a community, anatomists need to unite not for the survival of their profession but also to sustain high standards of humanism in their daily practices. They need to state that the dissection hall is not to be treated like many other facilities contributing to the university purse, but it is a monastery of scholarship where inquisitive and bright human minds have found a sanctuary for many centuries to learn the core values of life and mundane existence. People have entrusted anatomists with the post‐mortal remains of their loved ones with the hope that society will benefit from it.

**FIGURE 8:**
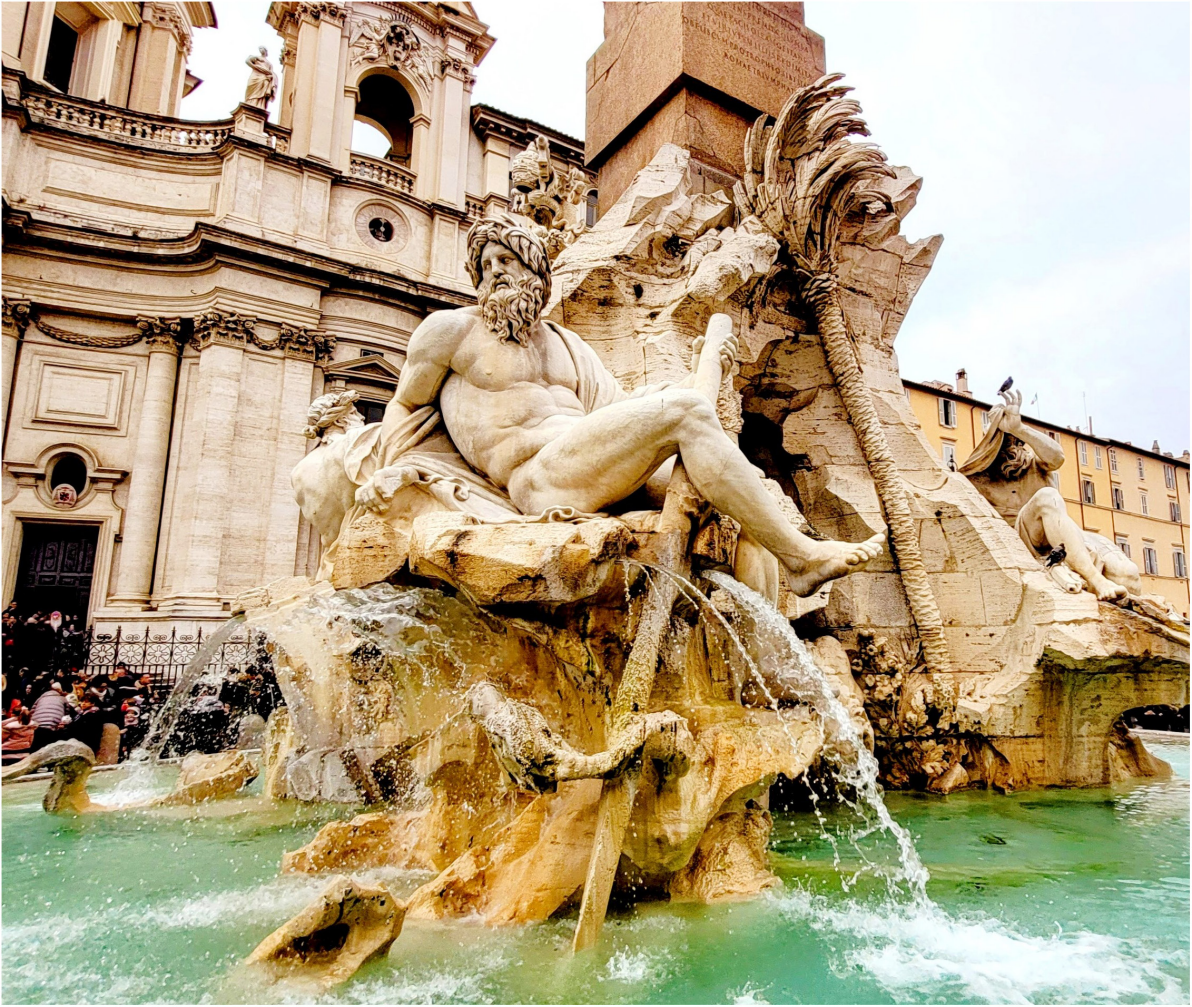
The sculpture at the famous Piazza Navona in Rome (Italy) displays excellent human anatomy with exquisite detailing of muscles. Figure from the personal collection of Sourav Bhattacharjee.

The dissection hall is not just a space but a stage where doctors are conceived, and philosophers are born (Figure 8). It belongs to medical students and teachers with no love lost between them. Together they have maintained medical education throughout the ages and, when necessary, have rectified their shortfalls with mutual dialogue. So, leave the dissection hall to the anatomists and their students as custodians of the discipline. Unless it speaks for these values, the future journey of humanism in anatomical practices, including ways of acquiring cadavers, will probably be lost in these silos of fuzzy documents and empty rhetoric.

There is an apparent hype regarding the growing use of platforms, like social media and virtual reality, in anatomy education (Hennessy & Smith, 2020). One of the main arguments favouring these alternate learning portals is skipping dissection, which will inevitably curtail the need for body acquisition. Some colleagues have also argued in support of teaching solely, or mostly, based on prosected materials. These alternative views bear merit as the need for hardship in maintaining high humanistic standards will be released if other forms of teaching and learning replace or facilitate cadaver‐based dissection. However, on a personal note, the authors would also argue that although these emerging tools are great for revising, especially before an exam, they still are not at par with cadaveric dissection, which remains more rewarding, satisfying, and informative (Franchi, 2020).

**FIGURE 9:**
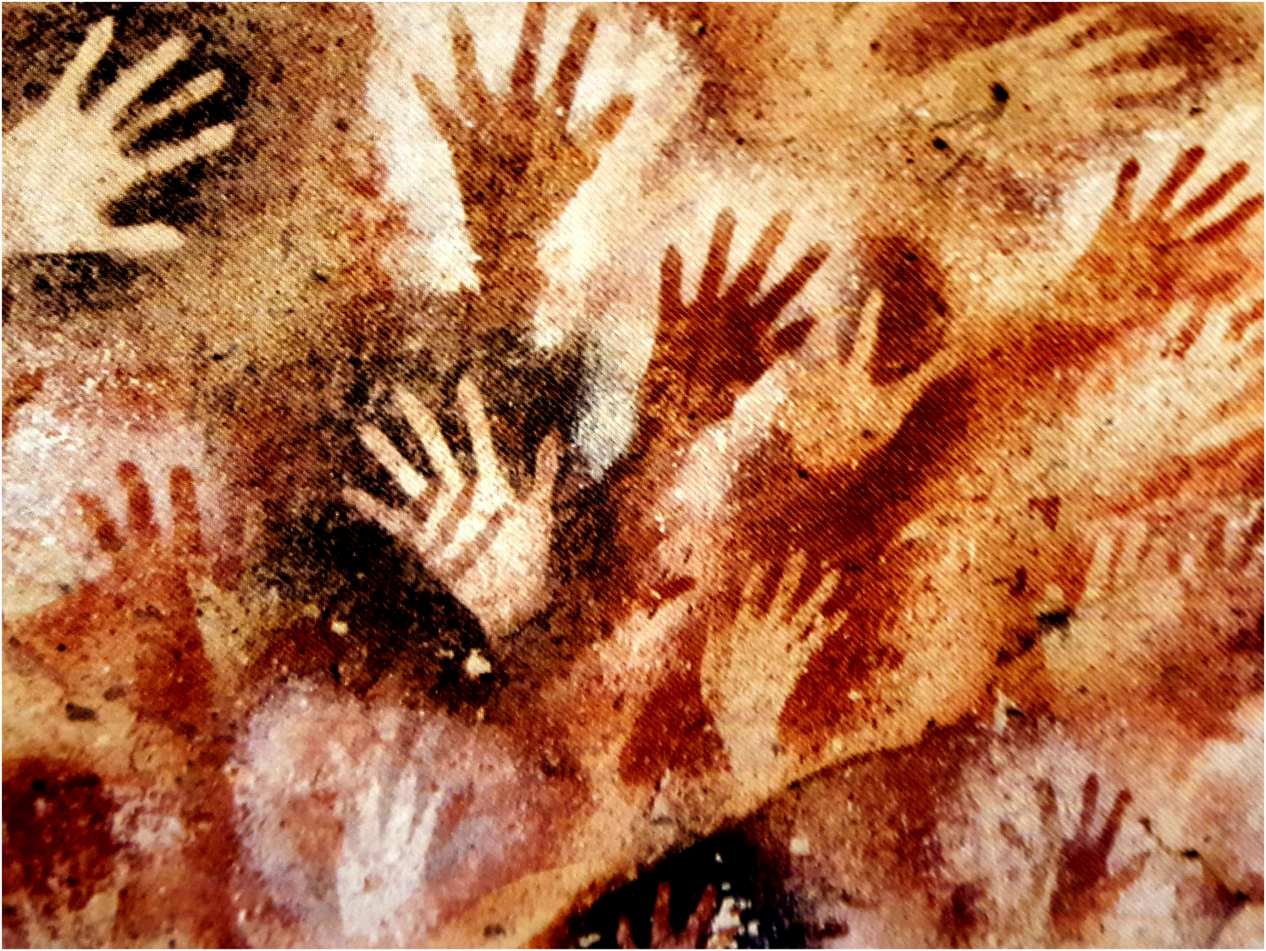
The hands waving a message for future generations as found at the Cave of the Hands (Cueva de las Manos) in Argentina. It was shown as an example of cave art at the Archaeological Museum of Alicante (Spain). Figure from the personal collection of Sourav Bhattacharjee.

To sum up, this account has tried to trace the evolution of humanistic standards in anatomical practices through the lens of cadaveric acquisition. An important message that distils out of the study is that how we perceive humanism within the dissection hall and the larger society, in general, keeps evolving as new value systems emerge to influence our lives, social customs, aspirations, and education. Thus, it is impossible to segregate anatomical studies or cadaveric acquisition, for that matter, from what is happening in our society, while good humanistic practices in anatomy can only be preserved in a society that nurtures humanistic beliefs (Figure 9). Anatomists hone the skills required to bridge current gaps between medicine and society. They carry the intellectual DNA of stalwarts, the likes of Renaissance giants, physicians, and researchers who, by their kindness, progressive thoughts, and actions, changed the discourse of history and gave hope to people. Anatomists must reach people, especially the marginalised sections, whom society has largely forgotten, and through their work and conviction, pump confidence that anatomists are not ready to compromise on their integrity.

Perhaps there is no better way of closing than quoting Tennyson:

“Tho’ much is taken, much abides; and tho’
We are not now that strength which in old days
Moved earth and heaven, that which we are, we are;
One equal temper of heroic hearts,
Made weak by time and fate, but strong in will
To strive, to seek, to find, and not to yield.”

## FUNDING INFORMATION

SB would like to thank UCD Research for funding.

## CONFLICT OF INTEREST STATEMENT

None declared.

## ETHICS STATEMENT

Not required.

## Supporting information


**File S1**.

**File S2**.

## Data Availability

No new data were shared in the manuscript. Further need for clarification, should it arise, may be obtained from or discussed with the corresponding author upon reasonable request.
