# Supplementary material for: The Sun Also Rises: Tracing the evolution of humanistic values in anatomy pedagogy and research, including cadaveric acquisition practices
Source: J Anat. 2023 Jul 31;243(6):1031–51. doi: 10.1111/joa.13938 (PMC10641044; doi:10.1111/joa.13938)
Supplement: Supplementary file 1 — File S1. [file JOA-243-1031-s001.pdf]

Anatomy Act, 1832

ANATOMY ACT 1832

C A P. LXXV.

An Act for regulating Schools of Anatomy. [1st *August* 1832.]

Secretary of State to grant Licences to practise Anatomy;

'Whereas a Knowledge of the Causes and Nature of sundry Diseases which affect the Body, and of the best Methods of treating and curing such Diseases, and of healing and repairing divers Wounds and Injuries to which the Human Frame is liable, cannot be acquired without the Aid of Anatomical Examination: And whereas the legal Supply of Human Bodies for such Anatomical Examination is insufficient fully to provide the Means of such Knowledge: And whereas, in order further to supply Human Bodies for such Purposes, divers great and grievous Crimes have been committed, and lately Murder, for the single Object of selling for such Purposes the Bodies of the Persons so murdered: And whereas therefore it is highly expedient to give Protection, under certain Regulations, to the Study and Practice of Anatomy, and to prevent, as far as may be such great and grievous Crimes and Murder as aforesaid;' be it therefore enacted by the King's most Excellent Majesty, by and with the Advice and Consent of the Lords Spiritual and Temporal, and Commons, in this present Parliament assembled, and by the Authority of the same, That it shall be lawful for His Majesty's Principal Secretary of State for the Time being for the Home Department in that Part of the United Kingdom called *Great Britain*, and for the Chief Secretary for *Ireland* in that Part of the United Kingdom called *Ireland*, immediately on the passing of this Act, or so soon thereafter as may be required, to grant a Licence to practise Anatomy to any Fellow or Member of any College of Physicians or Surgeons, or to any Graduate or Licentiate in Medicine, or to any Person lawfully qualified to practise Medicine in any Part of the United Kingdom, or to any Professor or Teacher of Anatomy, Medicine, or Surgery, or to any Student attending any School of Anatomy, on Application from such Party for such Purpose, countersigned by Two of His Majesty's Justices of the Peace acting for the County, City, Borough, or Place wherein such Party resides, certifying that, to their Knowledge or Belief, such Party so applying is about to carry on the Practice of Anatomy.

Secretary of State to appoint Inspectors of Schools of Anatomy;

II. And be it enacted, That it shall be lawful for His Majesty's said Principal Secretary of State or Chief Secretary, as the Case may be, immediately on the passing of this Act, or as soon thereafter as may be necessary, to appoint respectively not fewer than Three Persons to be Inspectors of Places where Anatomy is carried on, and at any Time after such first Appointment to appoint, if they shall see fit, One or more other Person or Persons to be an Inspector or Inspectors as aforesaid; and every such Inspector shall continue in Office for One Year, or until he be removed by the said Secretary of State or Chief Secretary, as the Case may be, or until some other Person shall be appointed in his Place; and as often as any Inspector appointed as aforesaid shall die, or shall be removed from his said Office, or shall refuse or become unable to act, it shall be lawful for the said Secretary of State or Chief Secretary, as the Case may be, to appoint another Person to be Inspector in his Room.

and to direct what District every Inspector shall superintend.

III. And be it enacted, That it shall be lawful for the said Secretary of State or Chief Secretary, as the Case may be, to direct what District of Town or Country, or of both, and what Places where Anatomy is carried on, situate within such District, every such Inspector shall be appointed to superintend, and in what Manner every such Inspector shall transact the Duties of his Office.

Inspectors to make Returns of Subjects removed for Anatomical Examination;

IV. And be it enacted, That every Inspector to be appointed by virtue of this Act shall make a Quarterly Return to the said Secretary of State or Chief Secretary, as the Case may be, of every deceased Person's Body that during the preceding Quarter has been removed for Anatomical Examination to every separate Place in his District where Anatomy is carried on, distinguishing the Sex, and, as far as is known at the Time, the Name and Age of each Person whose Body was so removed as aforesaid.

and to inspect Places where Anatomy is practised.

V. And be it enacted, That it shall be lawful for every such Inspector to visit and inspect, at any Time, any Place within his District, Notice of which Place has been given, as is herein-after directed, that it is intended there to practise Anatomy.

Salaries to Inspectors.

VI. And be it enacted, That it shall be lawful for His Majesty to grant to every such Inspector such an annual Salary, not exceeding One hundred Pounds, for his Trouble, and to allow such a Sum of Money for the Expences of his Office, as may appear reasonable; such Salaries and Allowances to be charged on the Consolidated Fund of the United Kingdom, and to be payable quarterly; and that an annual Return of all such Salaries and Allowances shall be made to Parliament.

Persons having lawful Custody of Bodies may permit them to undergo Anatomical Examination in certain Cases.

VII. And be it enacted, That it shall be lawful for any Executor or other Party having lawful Possession of the Body of any deceased Person, and not being an Undertaker or other Party intrusted with the Body for the Purpose only of Interment, to permit the Body of such deceased Person to undergo Anatomical Examination, unless, to the Knowledge of such Executor or other Party, such Person shall have expressed his Desire, either in Writing at any Time during his Life, or verbally in the Presence of Two or more Witnesses during the Illness whereof he died, that his Body after Death might not undergo such Examination, or unless the surviving Husband or Wife, or any known Relative of the deceased Person, shall require the Body to be interred without such Examination.

Provision in case of Persons directing Anatomical Examinations after their Death.

VIII. And be it enacted, That if any Person, either in Writing at any Time during his Life, or verbally in the Presence of Two or more Witnesses during the Illness whereof he died, shall direct that his Body after Death he examined anatomically, or shall nominate any Party by this Act authorized to examine Bodies anatomically to make such Examination, and if, before the Burial of the Body of such Person, such Direction or Nomination shall be made known to the Party having lawful Possession of the dead Body, then such last-mentioned Party shall direct such Examination to be made, and, in case of any such Nomination as aforesaid, shall request and permit any Party so authorized and nominated as aforesaid to make such Examination, unless the deceased Person's surviving Husband or Wife, or nearest known Relative, or any One or more of such Person's nearest known Relatives, being of Kin in the same Degree, shall require the Body to be interred without such Examination.

The Body not to be removed from the Place where such Person may have died without a Certificate.

IX. Provided always, and be it enacted, That in no Case shall the Body of any Person be removed for Anatomical Examination from any Place where such Person may have died, until after Forty-eight Hours from the Time of such Person's Decease, nor until after Twenty-four Hours Notice, to be reckoned from the Time of such Decease, to the Inspector of the District, of the intended Removal of the Body, or, if no such Inspector have been appointed, to some Physician, Surgeon, or Apothecary residing at or near the Place of Death, nor unless a Certificate stating in what Manner such Person came by his Death shall previously to the Removal of the Body have been signed by the Physician, Surgeon, or Apothecary who attended such Person during the Illness whereof he died, or if no such Medical Man attended such Person during such Illness, then by some Physician, Surgeon, or Apothecary who shall be called in after the Death of such Person to view his Body, and who shall state the Manner or Cause of Death according to the best of his Knowledge and Belief, but who shall not be concerned in examining the Body after Removal; and that in case of such Removal such Certificate shall be delivered, together with the Body, to the Party receiving the same for Anatomical Examination.

Professors, Surgeons, and others may receive Bodies for Anatomical Examination.

X. And be it enacted, That it shall be lawful for any Member or Fellow of any College of Physicians or Surgeons, or any Graduate or Licentiate in Medicine, or any Person lawfully qualified to practise Medicine in any Part of the United Kingdom, or any Professor, Teacher, or Student of Anatomy, Medicine, or Surgery, having a Licence from His Majesty's Principal Secretary of State or Chief Secretary as aforesaid, to receive or possess for Anatomical Examination, or to examine anatomically, the Body of any Person deceased, if permitted or directed so to do by a Party who had at the Time of giving such Permission or Direction lawful Possession of the Body, and who had Power, in pursuance of the Provisions of this Act, to permit or cause the Body to be so examined, and provided such Certificate as aforesaid were delivered by such Party together with the Body.

Such Persons to receive with the Body a Certificate as aforesaid, which shall be transmitted to the Inspector.

XI. And be it enacted, That every Party so receiving a Body for Anatomical Examination after Removal shall demand and receive, together with the Body, a Certificate as aforesaid, and shall, within Twenty-four Hours next after such Removal, transmit to the Inspector of the District such Certificate, and also a Return stating at what Day and Hour and from whom the Body was received, the Date and Place of Death, the Sex, and (as far as is known at the Time) the Christian and Surname, Age, and last Place of Abode of such Person, or, if no such Inspector have been appointed, to some Physician, Surgeon, or Apothecary residing at or near the Place to which the Body is removed, and shall enter or cause to be entered the aforesaid Particulars relating thereto, and a Copy of the Certificate he received therewith, in a Book to be kept by him for that Purpose, and shall produce such Book whenever required so to do by any Inspector so appointed as aforesaid.

Notice to be given to Secretary of State of Places where Anatomy is about to be practised.

XII. And be it enacted, That it shall not be lawful for any Party to carry on or teach Anatomy at any Place, or at any Place to receive or possess for Anatomical Examination, or examine anatomically, any deceased Person's Body after Removal of the same, unless such Party, or the Owner or Occupier of such Place, or some Party by this Act authorized to examine Bodies anatomically, shall, at least One Week before the first Receipt or Possession of a Body for such Purpose at such Place, have given Notice to the said Secretary of State or Chief Secretary, as the Case may be, of the Place where it is intended to practise Anatomy.

How Bodies are to be removed for Examination.

Provision for Interment.

XIII. Provided always, and be it enacted, That every such Body so removed as aforesaid for the Purpose of Examination shall, before such Removal, be placed in a decent Coffin or Shell, and be removed therein; and that the Party removing the same, or causing the same to be removed as aforesaid, shall make Provision that such Body, after undergoing Anatomical Examination, be decently interred in consecrated Ground, or in some public Burial Ground in use for Persons of that religious Persuasion to which the Person whose Body was so removed belonged; and that a Certificate of the Interment of such Body shall be transmitted to the Inspector of the District within Six Weeks after the Day on which such Body was received as aforesaid.

Persons described in this Act not to be liable to Punishment for having in their Possession Human Bodies.

XIV. And be it enacted, That no Member or Fellow of any College of Physicians or Surgeons, nor any Graduate or Licentiate in Medicine, nor any Person lawfully qualified to practise Medicine in any Part of the United Kingdom, nor any Professor, Teacher, or Student of Anatomy, Medicine, or Surgery, having a Licence from His Majesty's Principal Secretary of State or Chief Secretary as aforesaid, shall be liable to any Prosecution, Penalty, Forfeiture, or Punishment for receiving or having in his Possession for Anatomical Examination, or for examining anatomically, any dead Human Body, according to the Provisions of this Act.

Act not to prohibit post-mortem Examination.

XV. And be it enacted, That nothing in this Act contained shall be construed to extend to or to prohibit any post-mortem Examination of any Human Body required or directed to be made by any competent legal Authority.

So much of 9 G. 4. c. 31. as directs that the Bodies of Murderers may be dissected, repealed.

Such Bodies to be hung in Chains or buried, as the Court shall direct.

XVI. 'And whereas an Act was passed in the Ninth Year of the Reign of His late Majesty, for consolidating and amending the Statutes in *England* relative to Offences against the Person, by which latter Act it is enacted, that the Body of every Person convicted of Murder shall, after Execution, either be dissected or hung in Chains, as to the Court which tried the Offender shall seem meet; and that the Sentence to be pronounced by the Court shall express that the Body of the Offender shall be dissected or hung in Chains, whichsoever of the Two the Court shall order;' be it enacted, That so much of the said last-recited Act as authorizes the Court, if it shall see fit, to direct that the Body of a Person convicted of Murder shall, after Execution, be dissected, be and the same is hereby repealed; and that in every Case of Conviction of any Prisoner for Murder the Court before which such Prisoner shall have been tried shall direct such Prisoner either to be hung in Chains, or to be buried within the Precincts of the Prison in which such Prisoner shall have been confined after Conviction, as to such Court shall seem meet; and that the Sentence to be pronounced by the Court shall express that the Body of such Prisoner shall be hung in Chains, or buried within the Precincts of the Prison, whichever of the Two the Court shall order.

Limitation of Actions.

XVII. And be it enacted, That if any Action or Suit shall be commenced or brought against any Person for any thing done in pursuance of this Act, the same shall be commenced within Six Calendar Months next after the Cause of Action accrued; and the Defendant in every such Action or Suit may, at his Election, plead the Matter specially, or the General Issue Not guilty, and give this Act and the special Matter in Evidence at any Trial to be had thereupon.

Offences against this Act.

XVIII. And be it enacted, That any Person offending against the Provisions of this Act in *England* or *Ireland* shall be deemed and taken to be guilty of a Misdemeanor, and, being duly convicted thereof, shall be punished by Imprisonment for a Term not exceeding Three Months, or by a Fine not exceeding Fifty Pounds, at the Discretion of the Court before which he shall be tried; and any Person offending against the Provisions of this Act in *Scotland* shall, upon being duly convicted of such Offence, be punished by Imprisonment for a Term not exceeding Three Months, or by a Fine not exceeding Fifty Pounds, at the Discretion of the Court before which he shall be tried.

Interpretation of certain Words in this Act.

XIX. And in order to remove Doubts as to the Meaning of certain Words in this Act, be it enacted, That the Words "*Person and Party*" shall be respectively deemed to include any Number of Persons, or any Society, whether by Charter or otherwise; and that the Meaning of the aforesaid Words shall not be restricted although the same may be subsequently referred to in the Singular Number and Masculine Gender only.

Commencement of Act.

XX. And be it enacted, That this Act shall commence and take effect from and after the First Day of *August* in the present Year.

Act may be amended.

XXI. And be it enacted, That this Act may be altered or amended during the present Session of Parliament.
